# Total Marrow Irradiation as Part of Autologous Stem Cell Transplantation for Asian Patients with Multiple Myeloma

**DOI:** 10.1155/2013/321762

**Published:** 2013-09-08

**Authors:** Shih-Chiang Lin, Pei-Ying Hsieh, Pei-Wei Shueng, Hui-Ju Tien, Li-Ying Wang, Chen-Hsi Hsieh

**Affiliations:** ^1^Division of Medical Oncology and Hematology, Department of Internal Medicine, Far Eastern Memorial Hospital, New Taipei City 220, Taiwan; ^2^Department of Nursing, Oriental Institute of Technology, New Taipei City 220, Taiwan; ^3^Department of Biotechnology, School of Healthy Technology, Ming Chuan University, Taipei 111, Taiwan; ^4^Division of Radiation Oncology, Department of Radiology, Far Eastern Memorial Hospital, No. 21, Section 2, Nanya S. Road, Banqiao District, New Taipei City 220, Taiwan; ^5^School and Graduate Institute of Physical Therapy, College of Medicine, National Taiwan University, Taipei 100, Taiwan; ^6^Physical Therapy Center, National Taiwan University Hospital, Taipei 100, Taiwan; ^7^Department of Medicine, School of Medicine, National Yang-Ming University, Taipei 112, Taiwan; ^8^Institute of Traditional Medicine, School of Medicine, National Yang-Ming University, Taipei 112, Taiwan

## Abstract

To compare the outcomes of melphalan 200 mg/m^2^ (HDM200) and 8 Gy total marrow irradiation (TMI) delivered by helical tomotherapy plus melphalan 140 mg/m^2^ (HDM140 + TMI 8 Gy) in newly diagnosed symptomatic multiple myeloma (MM) Asian patients. Between 2007 and 2010, nine consecutive myeloma patients who were scheduled to undergo autologous stem cell transplantation (ASCT) were studied. The patients received three cycles of vincristine-adriamycin-dexamethasone (VAD) regimen as induction chemotherapy, and if they had a partial response, peripheral blood stem cells were collected by dexamethasone-etoposide-cyclophosphamide-cisplatin (DECP). In arm A, six patients received the HDM200. In arm B, three patients received HDM140 + TMI 8 Gy. In arm B, the neutropenic duration was slightly longer than in arm A (*P* = 0.048). However, hematologic recovery (except for neutrophils), transfusion requirement, median duration of hospitalization, and the dose of G-CSF were similar in both arms. The median duration of overall survival and event-free survival was similar in the two arms (*P* = 0.387). As a conditioning regiment, HDM140 + TMI 8 Gy provide another chance for MM Asian patients who were not feasible for HDM200.

## 1. Introduction

The outcome of autologous stem cell transplantation (ASCT) patients for newly diagnosed multiple myeloma (MM) is superior to that of patients receiving conventional chemotherapy [1–3]. Attal et al. [1] reported that 8 Gy total body irradiation (TBI) plus 140 mg/m^2^ intravenous melphalan (HDM140) improved the response rate and overall survival compared with conventional chemotherapy in patients with MM. The impact of complete response (CR) achievement has been shown with high dose preconditioning [1, 2]. To improve survival, the objective is to increase CR rates before autologous stem cell transplantation (ASCT); a total of 200 mg/m^2^ melphalan (HDM200) without TBI is an alternative method [2–4]. The Intergroupe Francophone du Mye'lome (IFM) 9502 trial compared the conditioning regimen 8 Gy TBI + HDM140 and HDM200 without TBI followed by ASCT [5]. The results revealed that HDM200 could be an alternative conditioning regimen for MM.

The complications cause by TBI have been reported. The pulmonary complications were concerned by TBI position, with beam energy (*P* = 0.02) [6] and the absence of lung shielding [7]. Helical tomotherapy (HT, Tomotherapy Hi-Art System, v. 3.2.2.35., TomoTherapy Inc., Madison, WI) is new CT-based rotational intensity modulated radiotherapy. Total marrow irradiation (TMI) with HT is designed to avoid the complications of TBI while achieving the effectiveness of TBI. Dosimetric studies showed reduced doses to adjacent critical normal organs reduced toxicity after TMI [8–10]. The advantages, acute toxicities, initial clinical experiences, and challenges of TMI were reported recently [10–12]. 

Recently, we reported the Asian experience with treating newly diagnosed MM patients with 8 Gy TMI by HT plus HDM140 [10]; patients subsequently received maintenance therapy with thalidomide [13] and dexamethasone. We found that HDM140 + TMI 8 Gy regimen was an acceptable conditioning regimen for MM patients. The preliminary outcomes were similar for Asians as for other races. In the current follow-up study, we compared the acute and early chronic toxicities, CR rates, very good partial response (VGPR) rates, and early results of progression-free survival (PFS) and overall survival (OS) in patients treated with HDM140 + TMI 8 Gy or HDM200 without TBI followed by ASCT.

## 2. Materials and Methods

### 2.1. Patient Characteristics

We enrolled nine consecutive myeloma patients who underwent ASCT at Far Eastern Memorial Hospital (diagnosed between 2007 and 2010). Eligibility criteria included age less than 65 years and symptomatic MM. Patients were excluded if they had the following: (1) stable stage I MM (Durie-Salmon classification [14]); (2) previous cytotoxic chemotherapy or radiotherapy; (3) severe abnormalities of cardiac, pulmonary, or hepatic function; or (4) serum creatinine levels >2 mg/dL. All patients gave informed consent, and the study was approved by the institutional ethics committee of the Far Eastern Memorial Hospital. 

### 2.2. Autologous Stem Cell Transplantation Regimen

**Figure 1:**
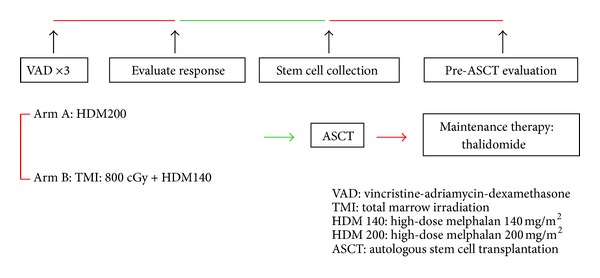
Study design profile.

The treatment protocol was modified from the Intergroupe Francophone du Myélome 9502 randomized trial [5]. Briefly, the patients received three cycles of the vincristine-adriamycin-dexamethasone (VAD) regimen, as in the trial (Figure 1). If they achieved a partial response (M-protein reduced by <50%), then they received one course of dexamethasone-etoposide-cyclophosphamide-cisplatin (DECP) with granulocyte colony-stimulating factor (G-CSF) mobilization. Two weeks later, peripheral blood stem cells (PBSCs) were collected. Stem cells were collected after G-CSF priming (10 *μ*g/kg/d) in steady state [15]. Daily apheresis was continued until at least 2 × 10^6^ CD34 cells per kilogram were collected. No CD34^+^ selection was performed. Two weeks after PBSC collection, the evaluation for ASCT was done. If cardiopulmonary, hepatic, and renal functions remained adequate, the patients received HDM140 + TMI 8 Gy or HDM200; the time from pre-HSCT evaluation to start of HDM140 + TMI 8 Gy or HDM200 (preconditioning) treatment was about 4 weeks. All of the patients received thalidomide for maintenance therapy after stem cell transplantation.

In arm A, HDM200 was administered for two days by infusion over 30 minutes. In arm B, patients received 8 Gy TMI by HT delivered in four fractions over a 4-day period (days 6, 5, 4, and 3) plus HDM140. HDM140 was administered for two days by infusion over 30 minutes, too. PBSC transplantation was performed on day 0. Hematopoietic growth factor support with G-CSF was provided on day 5 after transplantation until granulocyte recovery [5]. 

Thalidomide (50–200 mg/d) was started 100 days or later after TMI and was continued for 6 months following the achievement of complete remission, or for at least 12 months for patients with persistent evidence of residual disease [13].

### 2.3. Radiotherapy Technique

Details of the HT technique have been previously published [10]. Briefly, An AccuFix Cantilever Board (WFR/Aquaplast Corporation and Q-Fix Systems, LLC, Wyckoff, New Jersey, USA) with thermoplastic fixation or type-S thermoplastics head frame (MT-CHFN-C, Civco MedTec, Kalona, Iowa, USA) with mold care cushion was used for head and shoulder immobilization. A BlueBAG BodyFIX total body cushion system (Medical Intelligence, Schwabmünchen, Germany), which used a vacuum to produce a uniform pressure, was used to fix the main trunk and extremities in place. 

The radiotherapy was planned with patients in a supine position for head-first upper torso therapy and with feet-first lower extremity therapy. The planning CT images were performed using dual source CT (Siemens SOMATOM Definition, Siemens Healthcare, Erlangen, Germany) where three sets of images were acquired during normal breathing, inspiration, and shallow expiration for the upper torso. 

All of the CT images were sent to the Pinnacle^3^ Treatment Planning System (Philips Healthcare, Madison, Wisconsin, USA) for contouring. The clinical target volume (CTV) included the entire skeletal system. The margins for the planning target volume (PTV) were 0.8 cm for CTV extremities and 0.5 cm for all other bones of the CTV. The organs at risk (OAR) included the brain, optic nerves, lenses, eyes, parotid glands, oral cavity, thyroid gland, bilateral lungs, esophagus, heart, liver, spleen, pancreases, kidneys, bowel, bladder, and reproductive organs. After contouring the targets and critical organs, the images and structure set were sent to the Tomotherapy Hi-Art Planning Station for processing (Tomotherapy, Inc., Madison, Wisconsin, USA).

The prescription dose was 200 cGy per day (in 4 fractions) for a total dose of 800 cGy to the PTV. For the planning objective, at least 95% of the volume of PTV was to receive 800 cGy, with the mean dose to the OAR reduced to 50% of the prescribed dose. The field width, pitch, and modulation factor (MF) used for the treatment planning optimization were 2.5 cm, 0.32, and 3.0 for the upper torso and 5.0 cm, 0.4, and 2.0 for the lower extremities, respectively. 

### 2.4. Follow-Up and Response Criteria

Mandatory evaluations included physical assessment, routine hemogram, and comprehensive chemistry panel, serum protein electrophoresis every 3 months, and bone radiographs and bone marrow biopsies at 30 days and 6 and 12 months post-TMI or HDM200 and yearly thereafter. Toxicity of treatment was scored according to the Common Terminology Criteria for Adverse Events v3.0. Complete response (CR) was defined as the absence of serum and urinary M-protein and no more than 5% plasma cells on bone marrow. Very good partial response (VGPR) was defined as 90% or greater decrease in bone marrow plasma cells and blood M-protein levels. Partial response (PR) was defined as 50% or greater decrease in blood and bone marrow findings. Stable disease was defined as less than 25% decrease in blood and bone marrow findings for a minimum of 3 months. Progression was defined as greater than 25% increase in M protein, greater than 25% increase in bone marrow plasma cells, or new bone lesions [11].

### 2.5. Analysis

Descriptive statistical analyses were applied for patient and disease characteristics, treatment features, and toxicity. All analyses were performed using the SPSS, version 12.0 (SPSS, Chicago, IL, USA).

## 3. Results

### 3.1. Patient Population

Nine patients were enrolled between 2007 and 2010. In arm A, six patients received the common conditioning regimen of 200 mg/m^2^ melphalan. In arm B, three patients received the new regimen of 8 Gy TMI plus 140 mg/m^2^ melphalan. All of the patients received thalidomide for maintenance therapy after stem cell transplantation.

Patient characteristics for the nine patients are given in Table 1. The median age was 54 for arm A (range: 47–62) and 55 for arm B (range: 55–56). The majority of patients were treated for stage III disease. No patient had received prior radiotherapy. 

### 3.2. Response to Induction VAD Regimen and HDT

**Figure 2:**
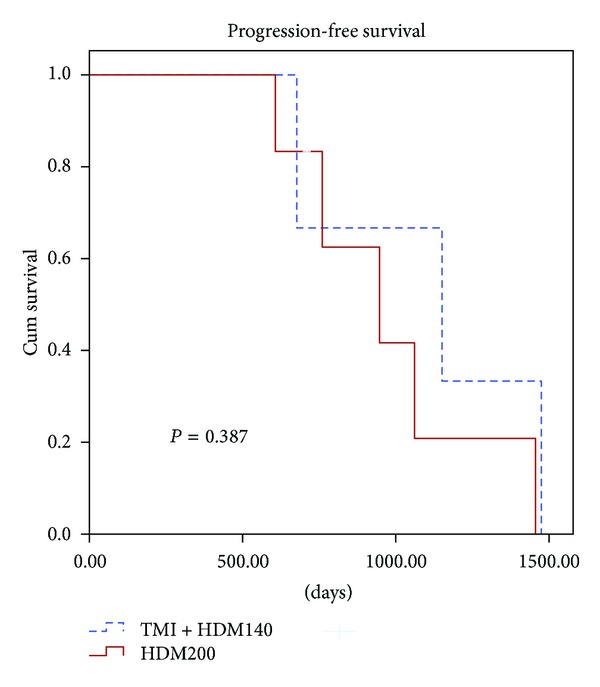
Cumulative (Cum) survival curves for patients treated with HDM200 or HDM140 + TMI 8 Gy. Curve for progression-free survival (according to treatment group) was illustrated. Cum survival: Cumulative survival.

In arm A, one patient achieved CR and the other one achieved VGPR before ASCT. In arm B, one patient achieved VGPR before ASCT. For arm A versus arm B, the CR rate to HDT was 1/6 versus 1/3; the VGPR rate to HDT was 4/6 versus 1/3; the median OS and PFS were 1223 versus 1556 days and 982 versus 1101 days, respectively (Table 2). The PFS was similar in the two arms (*P* = 0.387, Figure 2). Each group had one patient death due to disease progression.

### 3.3. Toxicities

Table 3 illustrates engraftment, hospitalization time, and transplantation-related toxicities. In arm B, the duration of neutropenia was one day longer than in arm A (*P* = 0.048, Table 3). Similar side effects were noted in the two groups. However, arm B experienced a shorter duration of thrombocytopenia, fewer platelet and red blood cell transfusions, and a shorter duration of intravenous antibiotic therapy than in arm A. 

## 4. Discussion

In newly diagnosed patients with MM treated with high-dose radiotherapy, the goal of the conditioning regimen is to achieve the best response rate with the least toxicity. The most widely used conditioning regimens are HDM200 and HDM140 + TBI [16]. The Intergroupe Francophone du Myélome 90 trial prospectively compared conventional chemotherapy with high-dose radiotherapy [1]. In this trial the conditioning regimen consisted of 8 Gy TBI plus HDM140. The CR rate after intensive therapy was 22%, and the response rate, event-free survival, and overall survival in patients with MM were improved. Furthermore, Moreau et al. [5] compared HDM200 and HDM140 + 8 Gy TBI as conditioning regimens for peripheral blood stem cell transplantation in patients with newly diagnosed MM. They reported that HDM200 could be an alternative conditioning regimen for MM.

 The pulmonary complications were statistically increased by prone and supine versus lateral TBI position (*P* = 0.02) and with 15 MV versus 9 MV beam energy (*P* = 0.02) [6]. A conditioning regimen of 12 Gy TBI in 6 daily fractions induces an interstitial pneumonitis incidence of about 11% in the absence of lung shielding [7]. Fatal interstitial pneumonitis using hyperfractionated TBI with standing position was still as high as 18% [17]. Compared with TBI technique, doses to the sensitive organs in TMI techniques were reduced by 15%–70% of the target dose [11, 18] or 1.7- to 7.5-fold reduction in median organ doses [8]. Somlo et al. [12] reported that the estimated median radiation dose to normal organs was 11% to 81% of the prescribed marrow dose. In our previous report, the dose reduction of TMI tomotherapy to various OARs of head, chest, and abdomen relative to TBI varied from 31% to 74%, 21% to 51%, and 46% to 63%, respectively [10]. The potential advantages, acute toxicities, initial clinical experiences, and challenges of this approach were reported recently [11]. The maximum tolerated dose for TMI was 1,600 cGy (200 cGy twice daily × 4 days) [12]. Under these doses, grade 3 or 4 nausea/emesis, fatigue, and metabolic abnormality were 2/22, 2/22, and 4/22, respectively [12]. Wong et al. [8] reported that grade 2 nausea and grade 1 emesis occurred only briefly on day 2 of TMI. Skin erythema, oral mucositis, esophagitis, and enteritis were not observed. In our previous experience of TMI treatment, one with grade 1 vomiting, two with grade 1 nausea, one with grade 1 mucositis, and three with grade 1 anorexia were noted [10]. These data hit the potential dosimetric and clinical advantages of TMI. Interestingly, the outcomes for the HDM200 and HDM140 + TMI 8 Gy conditioning regimens for MM are still inconclusive. In the current study, the CR and VGPR rates for arm A and arm B were 16.7% and 33.3%, 66.7% and 33.3%, respectively. The median OS and PFS were 1223 versus 1556 days and 982 versus 1101 days, respectively (Table 2). Additionally, the PFS rate was similar (Figure 2). With similar results as HDM200, HDM140 + TMI 8 Gy provide another chance to think about for MM Asian patients who were not feasible for HDM200. However, the lower relapse probability is noted in the patients receiving the higher dose of total body irradiation [19, 20]. In the current study, the dose of TMI is only 8 Gy. There still are spaces to titrate the radiation dose for the Asian in the future. 

Thalidomide has a broad spectrum of activities in multiple myeloma and is considered to improve event-free survival and OS [13, 21, 22]. The multiple effects include direct inhibition of myeloma cell growth and survival, direct stimulation of the cellular immune system, modulation of integrins compromising the adhesive interactions between the myeloma cells and bone marrow stroma, and antiangiogenic effects [23–25]. Previous studies demonstrated the impact of thalidomide maintenance of response after stem cell transplantation that improved event-free survival, complete response rate, and PFS rate [26–29]. In the current study, since the median PFS was similar between the groups, HDM140 + TMI 8 Gy appears to be as effective as HDM200 for Asians with MM.

Compared with HDM200, HDM140 + TBI had a greater toxicity regarding severe mucositis, duration of neutropenia and thrombocytopenia, number of red blood cell and platelet transfusions, number of days on antibiotics, and duration of hospitalization [5]. In the total therapy program, Barlogie et al. [30] used HDM200 for the second transplantation in responding patients. They found that HDM140 + 8.5- to 10-Gy TBI was quite toxic, and only 10% of the patients had no serious extramedullary toxicity. Treatment-related mortality was 2% with the second autotransplantation using HDM200 alone and rose to 5% with added TBI. In another retrospective evaluation, the duration of hospitalization was significantly reduced in the HDM200 cohort compared with HDM140 + TBI or HDM140 + busulfan cohort [16]. No treatment-related mortality was noted in the current study. In addition, there were no statistically significant differences between groups for engraftment, duration of thrombocytopenia, number of red blood cell and platelet transfusions, duration of antibiotic infusion days, and hospitalization time. HDM140 + TMI 8 Gy provided the similar effects and toxicities of a conditioning regimen as HDM200 dosing.

Moreau et al. [5] noted slower engraftment after HDM140 + TBI, despite the higher median number of CD34 cells infused than with HDM200 (7.3 versus 5, *P* = 0.03). Furthermore, compared with pregraft and normal control samples, patients treated with high-dose radiotherapy and autologous bone marrow transplantation revealed that conditioning regimens with TBI led more frequently to nonconfluent stromal layers [31]. Another study group analyzed fibroblast colony-forming units, the precursor compartment for the microenvironmental lineages essential to hematopoietic stem cell survival, proliferation, and differentiation [32]. The authors imply that the fibroblast damage could be due to the pretransplantation conditioning regimen. Bentley et al. [33] described patients who remained platelet and/or red cell transfusion dependent for 100 days or more after transplantation even after substantial neutrophil recovery. A significantly higher proportion of these patients had received TBI as part of their conditioning regimen. Together with these data, TBI may in some cases impair the early and late capacities of the marrow microenvironment to support transplanted stem cells. We noted similar median numbers of CD34 cells infused and numbers of red blood cell and platelet transfusions in both groups, except for the duration of neutropenia. These data suggest a potential benefit of TMI that could diminish impairment of the marrow microenvironment and provide similar results as HDM200. 

There are some limitations to our current study. First, the small case number and the retrospective study design make statistical conclusions highly tentative. However, in the current study, the percentages of stage III for both groups were more than 80%. With the similar progression-free survival days for both groups, HDM 140 + TMI regimen provided another chance for multiple myloma Asian patients who could not tolerate HDM200 regimen. Second, the follow-up time was short so that late effects are insufficiently addressed.

## 5. Conclusions

Based on these preliminary data, we are encouraged by the clinical results of HDM140 + TMI 8 Gy treatment of Asian patients with MM. This regimen has manageable toxicity and is at least as effective of a conditioning regimen as HDM200. Further evaluation is needed to learn what longer-term effect the regimen will have on disease control. Additionally, long-term followup is needed to characterize the long-term toxicities and assess the full impact in this new approach to ASCT pretreatment in Asian patients with MM. 

## Figures and Tables

**Table 1:** Main characteristics at diagnosis of the nine patients according to treatment group.

	Arm A (*n* = 6)	Arm B (*n* = 3)	*P*
Age	54 (47–62)	55 (55-56)	1.000
Gender			
Female	3 (50%)	2 (67%)	0.595
Male	3 (50%)	1 (33%)	
Durie-Salmon stage			
2A	1 (17%)	0	0.643
3A	3 (50%)	3 (100%)	
3B	2 (33%)	0	
M component			
IgG	4 (67%)	3 (100%)	0.417
LCD	2 (33%)	0	
Hemoglobin	6.6 (5.5–12.5)	9.6 (8.4–11.1)	1.000
Serum calcium	8.75 (7.6–9.4)	9.6 (8.7–11.2)	1.000
Serum creatinine	1.74 (0.62–3.86)	0.93 (0.8–1.1)	0.524
Serum B2-microglobulin	2637 (1890–3033)	2143 (1515–3376)	1.000

**Table 2:** Response to induction vincristine-adriamycin-dexamethasone (VAD) regimen and high dose therapy (HDT).

	Arm A	Arm B	*P*
Median no. of course of VAD (range)	3 (3-4)	3 (3)	1.000
Response to VAD			1.000
CR	1	0	
VGPR	1	1	
PR	4	2	
Median no. of CD34 (10^6^/kg) infused (range)	6.75 (3.6–9.14)	4.9 (4.12–6.21)	0.167
Response to HDT			1.000
CR	1	1	
VGPR	4	1	
PR	1	1	
Toxic death	0	0	1.000
Death due to disease progress	1	1	1.000
Overall survival, day (median)	1223 (709–1659)	1566 (737–2160)	0.515
Progression-free survival, day (median)	982 (607–1456)	1101 (677–1475)	0.387

CR: complete response; VGPR: very good partial response; PR: partial response.

**Table 3:** Engraftment, hospitalization time, and transplantation-related toxicity.

	Arm A	Arm B	*P*
Dose of G-CSF (median)	2675 (18 K–36 K)	2600 (15 K–39 K)	1.000
Duration of neutropenia, day (median)	9.5 (6–19)	10.7 (10-11)	0.048*
Duration of thrombocytopenia, day (median)	16.2 (7–23)	11.3 (10–13)	0.167
No. of platelet transfusions (median)	42 (24–72)	24 (12–36)	1.000
No. of red blood cell transfusions (median)	2.3 (0–8)	1.3 (0–4)	1.000
Duration of hospitalization, day (median)	28.2 (26–32)	29.7 (28–31)	0.226
Duration of intravenous antibiotics, day (median)	5.2 (0–13)	3.3 (0–10)	1.000

*: *P* value < 0.05.
